# Investigating Brain Connectomic Alterations in Autism Using the Reproducibility of Independent Components Derived from Resting State Functional MRI Data

**DOI:** 10.3389/fnins.2017.00459

**Published:** 2017-09-08

**Authors:** Mohammed A. Syed, Zhi Yang, Xiaoping P. Hu, Gopikrishna Deshpande

**Affiliations:** ^1^Computer Science and Software Engineering Department, Auburn University Auburn, AL, United States; ^2^Shanghai Key Laboratory of Psychotic Disorders, Shanghai Mental Health Center, Shanghai Jiao Tong University School of Medicine Shanghai, China; ^3^Brain Science and Technology Research Center, Shanghai Jiao Tong University Shanghai, China; ^4^The Department of Bioengineering, University of California, Riverside Riverside, CA, United States; ^5^The Department of Electrical and Computer Engineering, AU MRI Research Center, Auburn University Auburn, AL, United States; ^6^The Department of Psychology, Auburn University Auburn, AL, United States; ^7^The Alabama Advanced Imaging Consortium at Auburn University, University of Alabama Birmingham Auburn, AL, United States

**Keywords:** autism, fMRI, independent components, reproducibility, clustering

## Abstract

**Significance:** Autism is a developmental disorder that is currently diagnosed using behavioral tests which can be subjective. Consequently, objective non-invasive imaging biomarkers of Autism are being actively researched. The common theme emerging from previous functional magnetic resonance imaging (fMRI) studies is that Autism is characterized by alterations of fMRI-derived functional connections in certain brain networks which may provide a biomarker for objective diagnosis. However, identification of individuals with Autism solely based on these measures has not been reliable, especially when larger sample sizes are taken into consideration.

**Objective:** We surmise that metrics derived from Autism subjects may not be highly reproducible within this group leading to poor generalizability. We hypothesize that functional brain networks that are most reproducible within Autism and healthy Control groups separately, but not when the two groups are merged, may possess the ability to distinguish effectively between the groups.

**Methods:** In this study, we propose a “discover-confirm” scheme based upon the assessment of reproducibility of independent components obtained from resting state fMRI (discover) followed by a clustering analysis of these components to evaluate their ability to discriminate between groups in an unsupervised way (confirm).

**Results:** We obtained cluster purity ranging from 0.695 to 0.971 in a data set of 799 subjects acquired from multiple sites, depending on how reproducible the corresponding components were in each group.

**Conclusion:** The proposed method was able to characterize reproducibility of brain networks in Autism and could potentially be deployed in other mental disorders as well.

## Introduction

Autism Spectrum Disorder (ASD) is characterized as a developmental disability leading to significant social, communication and behavioral challenges (American Psychiatric Association, 2013). In 2010, an estimate from the Autism and Developmental Disabilities Monitoring (ADDM) Network involving 11 sites revealed that 14.7 per 1,000 or 1 in 68 children aged 8 years were affected by this disorder (Wingate et al., 2012; Baio, 2014). In addition, this study discovered that one in 54 males and one in 252 females in the ADDM communities had Autism. These disorders have been found to be very heritable (Muhle et al., 2004). In addition, approximately 18.7% of infants with at least one older sibling with Autism developed this disorder (Ozonoff et al., 2011). Given the societal implications of Autism, early diagnosis and intervention has become paramount. However, Autism is currently diagnosed using behavioral tests which can be subjective. Consequently, objective non-invasive biomarkers of Autism are being actively researched.

In order to find objective biomarkers of Autism, studies have used information from brain imaging techniques such as structural Magnetic Resonance Imaging (MRI). Ecker et al. (2010) used a multiparameter classification approach involving a support vector machine (SVM) to characterize the structural pattern of gray matter anatomy in adults with ASD and examined a set of five morphological parameters such as volumetric and geometric features at each spatial location on the cortical surface to discriminate between people with ASD and controls. Jiao et al. (2010) built diagnostic models for ASD based upon regional thickness measurements extracted from surface-based morphometry (SBM) and compared these models to diagnostic models based on volumetric morphometry using four machine learning techniques: support vector machines (SVM), multilayer perceptrons (MLPs), functional trees (FTs), and logistic model trees (LMTs). Voxel-based morphometry along with a multivariate pattern analysis approach was used by Uddin et al. (2011) to determine multiple brain regions showing atypical structural organization in children with Autism. Calderoni et al. (2012) examined whole brain volumes of female subjects with ASD using mass-univariate and pattern classification approaches. Sato et al. (2013) extracted individual subject features from inter-regional thickness correlations based on structural MRI which were later used in a machine learning framework to obtain subject level prediction of severity scores based upon neurobiological criteria rather than behavioral information. Libero et al. (2015) examined multiple brain imaging modalities to investigate the neural architecture in the same set of subjects using techniques such as decision tree classification analysis. Functional (as opposed to structural) MRI has been used in several studies on Autism as well. The feasibility of a functional MRI connectivity diagnostic assay for Autism was investigated by Anderson et al. (2011) after obtaining pairwise functional connectivity measurements from a lattice of 7,266 regions of interest covering the entire gray matter and using a single resting state blood oxygen level-dependent scan of 8 min for classification in each subject. Coutanche et al. (2011) used data from an fMRI study of the neural basis for face processing in subjects with ASD to illustrate that multi-voxel pattern analysis (MVPA) may provide a sensitive functional biomarker of clinical symptom severity. Wang et al. (2012) used a multi-scale clustering methodology known as “data cloud geometry” to extract functional connectivity patterns from fMRI for the recognition of ASD subjects by applying it to correlation matrices of 106 regions of interest (ROIs) in subjects with ASD and controls. Deshpande et al. (2013) used supervised machine learning and fMRI to show alterations in causal connectivity in the brain could serve as a potential non-invasive neuroimaging signature for Autism. Nielsen et al. (2013) also used pairwise functional connectivity measurements from a lattice of 7,266 regions of interest covering the gray matter for 964 subjects to conclude that multisite classification based on functional connectivity derived from resting state fMRI of Autism performed better than chance using a simple leave-one-out classifier. Maximo et al. (2013) used regional homogeneity and local density approaches at different spatial scales and examined local connectivity in ASD, while Supekar et al. (2013) showed hyper-connectivity in a sample of relatively younger Autistic kids using resting state fMRI. The common theme emerging from the studies mentioned above is that Autism is characterized by altered functional connectivity in certain brain networks and that characterizing this appropriately using MRI-based methods may provide a biomarker for objective diagnosis.

**Figure 1:**
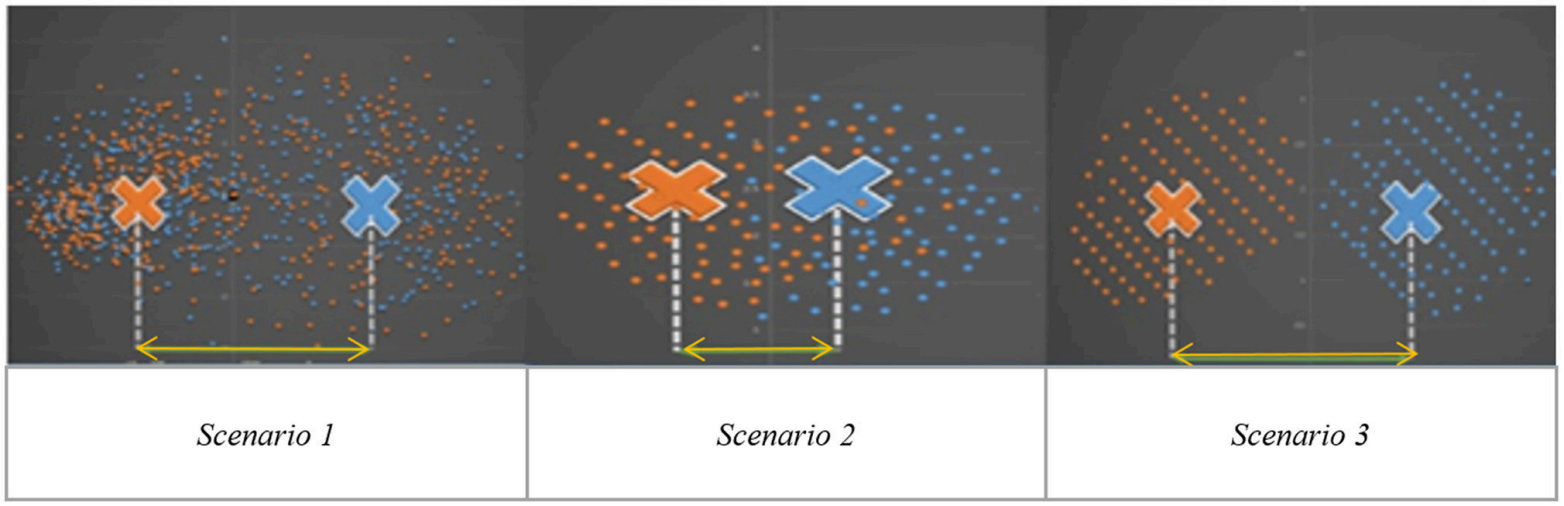
Imaginary feature space showing three different scenarios while discriminating between two groups (indicated by two colors orange and blue): Autism and Healthy Control. Scenario 1: Significant group difference in means, say, x. Scenario 2: Non-significant difference in group means. Scenario 3: Significant mean difference comparable to x in scenario 1.

Independent Component Analysis (ICA) is a blind source separation technique which is commonly employed for extracting brain networks involving spatially distributed regions with similar/correlated temporal activity (Bell and Sejnowski, 1995), especially in the baseline resting state. Consequently, it has been applied to investigate altered brain networks in Autism using fMRI. Specifically, Von von dem et al. (2013) employed ICA to demonstrate that individuals with Autism had reduced functional connectivity within the Default Mode Network (DMN), an important resting state brain network (Greicius et al., 2003). Assaf et al. (2010) studied the role of altered functional connectivity of the default mode sub-networks in ASDs using short resting fMRI scans and ICA. In spite of these studies showing reduced connectivity in certain brain networks in Autism, identification of individuals with Autism solely based on these measures has not been reliable, especially in samples of large sizes (Nielsen et al., 2013). We surmise that one major factor contributing to this state of affairs may be that metrics derived from Autism and/or Control subjects may not be highly reproducible within their respective group. Consequently, such metrics have poor generalizability, leading to lower cluster purities. Therefore, in this paper, we hypothesize that functional brain networks which are most reproducible separately within Autism and healthy Control groups, but not reproducible when both groups are merged, may possess the ability to effectively discriminate between the groups. The basis for this hypothesis is illustrated in Figure 1 which shows an imagined feature space where we want to discriminate between the two groups (Autism and healthy Control). Please note that Figure 1 has not been drawn to scale and is an illustrative schematic.

Scenario-1 corresponds to the situation wherein the two groups have significantly different means (say, x) in the feature space. However, within each group, the features have poor reproducibility (i.e., they are more scattered in the feature space), likely due to the heterogeneity of the disorder. Therefore, even if the group means are statistically separated, such features will give poor cluster purity. Scenario-2 is a situation where there is no significant difference between means, but the features are reproducible in the combined group (i.e., Autism + Control group), i.e., they are less scattered in the feature space even when both groups are combined. These two scenarios indicate that features which are highly reproducible separately in each group but are not reproducible in the combined (Autism + Control) group are likely to provide purer clusters while discriminating between the Autism and Control groups. In the third scenario, the features are not only statistically separated between the groups (with the difference between the group means comparable to “x” in Scenario-1), but also reproducible within each group, i.e., less scattered in feature space within each group. In order to test our hypothesis, traditional ICA-based characterization of the functional brain needs to be modified such that reproducibility information is considered while choosing independent components. Therefore, we propose a methodology involving assessment of reproducibility of independent components, followed by clustering analysis of such components for evaluating their discriminability between groups in an unsupervised way. Accordingly, we applied a recently introduced algorithm, “generalized Ranking and Averaging Independent Component Analysis by Reproducibility” (gRAICAR, https://github.com/yangzhi-psy/gRAICAR) (Yang et al., 2012), which can provide independent components that are highly reproducible within a given group of subjects. This technique is an extension of a framework previously developed for single subject analysis called Ranking and averaging independent component analysis by reproducibility—RAICAR (Yang et al., 2008) and has been successfully used in a number of applications (Yang et al., 2014a,b). In this work, gRAICAR was applied to Autism Brain Imaging Data Exchange (ABIDE) data (Di Martino et al., 2014) to estimate the independent components which are most reproducible, in Autism and Control groups, respectively, but not reproducible in the combined group. We input the spatial maps of such independent components into a k-means clustering algorithm and determined the purity of each cluster with respect to the *a priori* clinical diagnosis received by subjects.

## Materials and methods

### Composition of the subject sample

We utilized resting-state functional magnetic resonance imaging (R-fMRI) data from 799 individuals provided by Autism Brain Imaging Data Exchange (ABIDE). The data we used had 392 individuals with Autism spectrum disorders and 407 age- and sex-matched typical controls (TCs). These data came from 13 different imaging sites and included 700 male and 99 female subjects (Table 1) between 7 and 64 years of age. Data were fully anonymized wherein all 18 HIPAA (Health Insurance Portability and Accountability)-protected health information identifiers were removed. Data contributions were based on studies approved by the local Institutional Review Boards. Detailed information regarding the imaging data sets and associated phenotypic protocols can be found at http://fcon_1000.projects.nitrc.org/indi/abide. Data acquisition parameters and individual site details are also available on this web site.

**Table 1:** Institute names used in our study from ABIDE data and subject distribution by diagnosis code, Autism and Control.

**ID**	**Institute name**	**Autism**	**Control**
1	California Institute of Technology	19	19
2	Kennedy Krieger Institute	22	33
3	University of Leuven	29	35
4	Ludwig Maximilians University Munich	24	33
5	Oregon Health and Science University	12	14
6	University of Pittsburgh	30	27
7	Social Brain Lab UMC Groningen NIN	15	15
8	San Diego State University	14	21
9	Stanford University	17	18
10	Trinity College Dublin	24	25
11	University of California Los Angeles	62	47
12	University of Michigan	68	77
13	University of Utah	56	43

### Pre-processing

We first converted the data downloaded from ABIDE database, which was in DICOM format, to Neuroimaging Informatics Technology Initiative (NIfTI) format. In order to complete the first step, we used dcm2nii software which is freely available at http://www.mccauslandcenter.sc.edu/mricro/mricron/dcm2nii.html.

In the next step, we used a combination of Data Processing Assistant for Resting-State fMRI (Yan and Zang, 2010; DPARSF, http://www.restfmri.net), which is a plug-in software based on Statistical Parametric Mapping or SPM (http://www.fil.ion.ucl.ac.uk/spm), and uses functionality from Resting-State fMRI Data Analysis Toolkit (REST 1.7) (Song et al., 2011), both of which run on MATLAB. DPARSF was used to perform realignment of 3D brain volumes at each instant relative to the initial volume using 6-parameter rigid body registration, normalization to MNI (Montreal Neurological Institute) template using nonlinear warping, spatial smoothing using a Gaussian kernel with full width at half maximum of 4 mm × 4 mm × 4 mm, de-trending using linear polynomial and temporal band-pass filtering using the frequency range of 0.01–0.1 Hz.

Four Dimensional NIfTI-1 format images (http://nifti.nimh.nih.gov/nifti-1) from the pre-processing described above were then used in FMRIB Software Library v5.0 (Woolrich et al., 2009; Jenkinson et al., 2012) (FSL by Analysis Group, FMRIB, Oxford, UK) to obtain a set of independent components for each subject using Multivariate Exploratory Linear Optimized Decomposition into Independent Components (MELODIC) algorithm (Beckman and Smith, 2004; Beckman et al., 2005). FSL provides analysis tools for fMRI, MRI and DTI brain imaging data, including ICA for decomposing single or multiple 4D data sets into linearly independent spatial components. More information on MELODIC is available at http://fsl.fmrib.ox.ac.uk/fsl/fslwiki/MELODIC. We used the MELODIC analysis tool to perform standard 2D spatial ICA on each subject resulting in time courses (one per component) in the mixing matrix and spatial maps (one per component). The number of components for each subject was determined by MELODIC through automatic dimensionality estimation. We saved MELODIC results for each subject and used them in the algorithm we describe in the following section, for finding reproducible independent components.

### gRAICAR algorithm

The dataset from subject *s (s*=*1,2,…,n)* can be represented as a *t*_*s*_ × *v*_*s*_ matrix, ***M***_*t*_, where *t*_*s*_ represents the number of time points and *v*_*s*_ the number of voxels. The data matrix, ***M***_*s*_, can be decomposed into *c*_*S*_ independent components (ICs) in spatial domain *s*_*s*_(*c*_*s*_ × *v*_*s*_ *matrix*, ***M***_*c*_) and their corresponding mixing time courses *a*_*s*_(*t*_*s*_ × *c*_*s*_ matrix, ***M***_*a*_).

**Figure 2:**
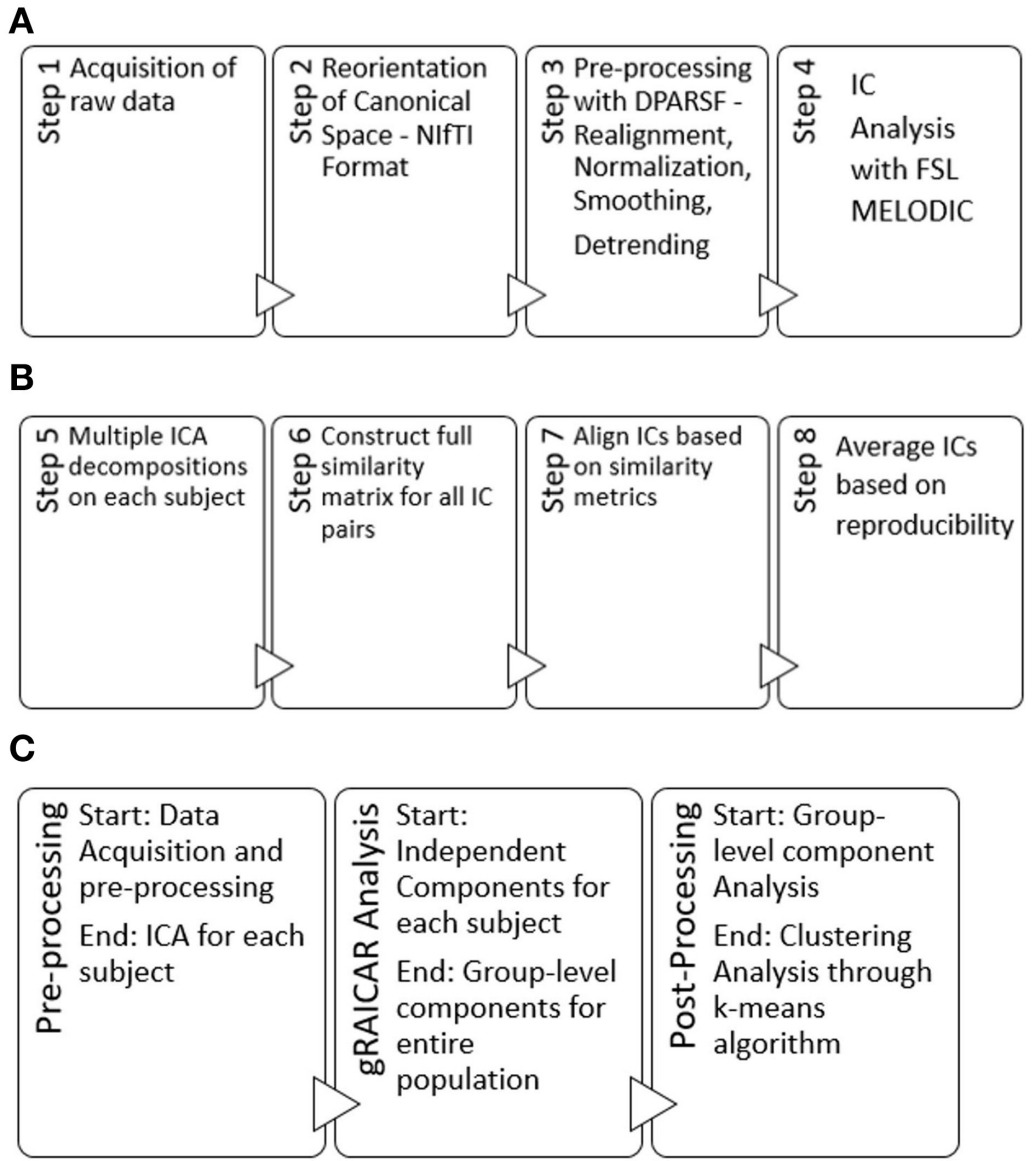
**(A)** Steps used in our pre-processing methodology have been summarized. Step 1 involved obtaining raw multi-site data for each subject in .nii.gz format. Step 2 involved converting raw data to NIfTI format which resulted in pairs of header and image files (hdr/.img) for each subject, using dcm2nii software. Step 3 involved processing data for each subject using a combination of MATLAB, DPARSF, SPM and REST to obtain a 4D .nii file for each subject based upon the input .hdr/.img files. Step 4 included the processing of 4D files obtained from step 3 in FSL—MELODIC using group ICA for each site leading to independent components or ICs to be used in our algorithm. Step 4 was the last step in our pre-processing methodology. **(B)** Schematic illustrating the 4 steps (5–8) of the gRAICAR algorithm once the pre-processing is complete. **(C)** Workflow of our analysis.

**Figure 3:**
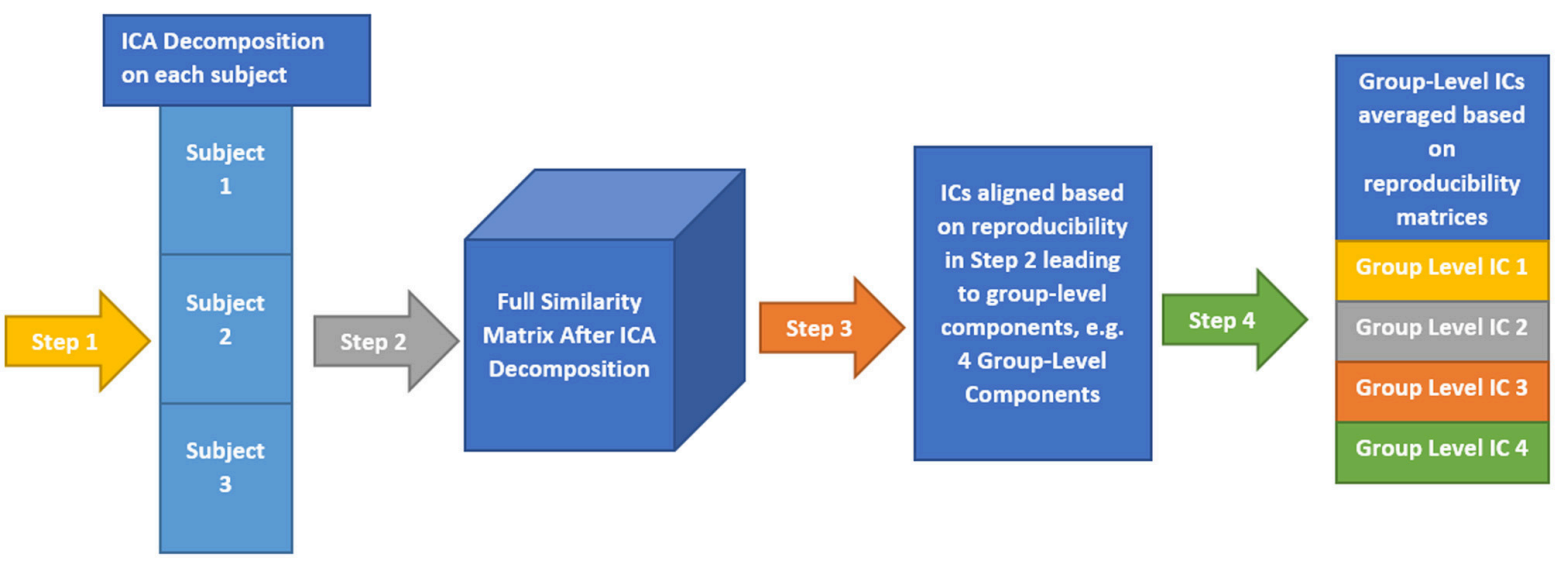
This illustrates the implementation of gRAICAR algorithm using 3 subjects with 3 subject-level components each as an example. Step 1 involves multiple ICA realizations for each subject. Step 2 is used to create a full similarity matrix (FSM). In step 3, ICs are aligned based upon similarity metrics. In step 4, ICs are averaged based upon reproducibility matrices.

Here, we provide a brief overview of gRAICAR and the readers are referred to the original paper by Yang et al. (2012) for a more comprehensive description. The algorithm contains four stages of processing as summarized in Figure 2. (1) The first step involved performing ICA decomposition *d* times for each subject using random initial values leading to *d* × *n* realizations where *n* is the number of subjects. We refer to these realizations as *REs*. In our study, *RE*_*ij*_ refers to the ICs from *jth* realization of subject *i*. (2) In its second step, a full similarity matrix (FSM) that had relational measures between all *REs* was constructed. Similarity between two REs in this algorithm was quantified by using normalized mutual information or *NMI*. (3) In the third step, *REs* that were found to be highly reproducible across subjects and ICA realizations were extracted and aligned. Two related *REs* were considered as individual-level components with the same underlying group-level component or an aligned component (*AC*). For each *AC*, the algorithm generated a *dn* × *dn* reproducibility matrix, ***M***_*R*_, within which *NMIs* between all pairs of *REs* pertaining to the *AC* were collected. (4) In the fourth step, we aligned *ACs* to obtain group-level component maps and examined the inter-subject consistency. While Figure 2B illustrates the algorithm in general terms, we demonstrate the specific implementation of this algorithm for an example of three subjects in Figure 3.

We applied the gRAICAR algorithm separately to Autism, Control and Combined groups. The first step involved performing ICA decomposition *d* (~5,000 for this study) times for each subject using random initial values leading to *d* × *n* realizations where *n* is the number of subjects. Specifically, *d* × *n* or *d* × 392 realizations of ICs for Autism group, *d* × 407 realizations in the TC group and *d* × 799 realizations in the combined group were obtained. These ICA realizations are named *REs* and *RE*_*ij*_ is used to denote the set of ICs from the *j*th realization of the *i*th subject.

**Figure 4:**
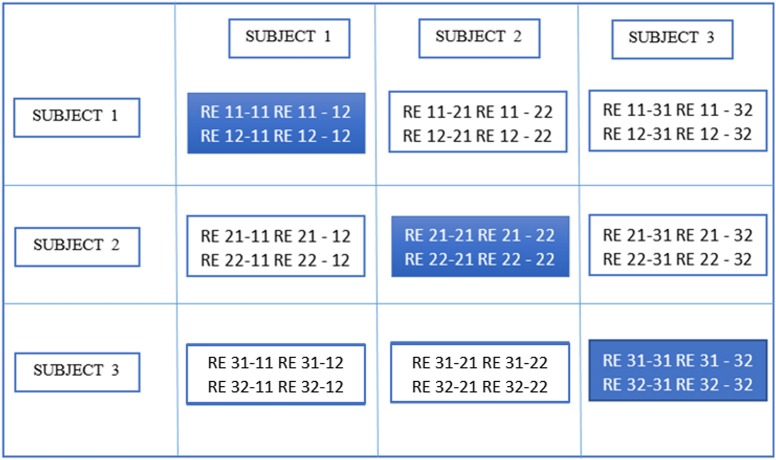
Block structure of full similarity matrix (**FSM**) based upon our example with 3 subjects and 2 sets of subject-level components (RE, a set of ICs produced after two ICA decompositions). In this example, each block shows similarity between 2 pairs of REs from the same subject or two different subjects. Also, RE 11-31 indicates inter-subject similarity between RE 1 of subject 1 and RE 1 of subject 3. The first digit in 11 is an index to the subject whereas the second is an index to the RE. Solid colored, on the diagonal blocks indicate similarity within the components of a subject or intra-subject similarity whereas off diagonal ones indicate that between the components of two different subjects.

In the second stage of gRAICAR, we constructed a full similarity matrix (***FSM***). This matrix has relational measures between all *REs*. Block structure of the ***FSM*** represents subject blocks (*SBs*) that in turn represent subject-wise relationships. Elements within these blocks can determine similarity between *REs* from the same subject or pairs of *REs* from different subjects depending on the location of the block. In these *SBs*, there are *d* × *d* realization blocks (*RBs*) providing pair-wise similarity between *REs* from different ICA realizations. This similarity between two *RBs* was quantified by using normalized mutual information or *NMI* (Pluim et al., 2003). *NMI* is one if two variables are identical and zero if they are statistically independent, revealing higher order statistical similarity as opposed to second order similarity expressed by correlation or covariance (Maes et al., 1997). *NMI* between each IC pair were computed using mutual information using the algorithm proposed by Kraskov et al. (2004). *NMIs* were further standardized within an *RB*, resulting in standardized *NMI (SNMI)*. In order to demonstrate the technical underpinnings and rationale for this stage, Figure 4 presents the block structure of ***FSM*** with 3 artificial subjects and two ICA realizations each (i.e., *d* = *2, n* = *3*).

Blocks marked with solid lines are called Subject blocks or *SBs*. Off diagonal *SBs* indicate the similarity between pairs of *REs* from different subjects while the ones on the diagonal reflect that from the same subject. The *RB* is represented as a *c*_*i*_ × *c*_*m*_ matrix, ***M***_*RB*_, with *R*_*ij*−*mk*_ reflecting the similarity between *RE*_*ij*_ and *RE*_*mk*_ *(i, m: 1,2,…n, j,k: 1,2,…d)*. *NMI* as mentioned above for two *REs* can now be calculated as:

(1) $$\begin{array}{l} R_{ij-mk}\left[y,\;z\right]=NMI\left(RE_{y}\in RE_{ij\;},\;RE_{z}\in RE_{mk\;}\right)\\ \;=\frac{H\left(RE_{y}\right)+H\left(RE_{z}\right)}{H\left(RE_{y},\;RE_{z}\right)}-1 \end{array}$$

where *H*(*RE*_*y*_), *H*(*RE*_*z*_), *H*(*RE*_*y*_, *RE*_*z*_) represent entropies of random variables, *RE*_*y*_, and *RE*_*z*_, and the mutual entropy between them *(1* ≤ *y* ≤ *C*_*i*_*, 1* ≤ *z* ≤ *C*_*m*)_. The *NMIs* were further normalized in the alignment procedure using,

(2) $$\begin{array}{l} R_{ij-mk}\left[y,z\right]\\ =\;\frac{R_{ij-mk}\;\left[y,\;z\right]-mean\;\left(R_{ij-mk}\;\left[y,*\right]\;\cup \;R_{ij-mk}\;\left[*,z\right]\right)}{std\;\left(R_{ij-mk}\;\left[y,.\right]\;\cup \;R_{ij-mk}\;\left[.,z\right]\right)}, \end{array}$$

In Equation (2), ^*^ represents all *NMI* values in row *y* or column *z* of the *RB*. This standardized *NMI* or *SNMI* can be used to calculate the specificity of individual similarity values associated with a given *RE* within the *RB*. The diagonal *RBs* are normally set to zero since they represent identity matrices and are therefore not of interest.

We then extracted highly reproducible *REs* across subjects and ICA realizations and aligned them in the third stage of the gRAICAR algorithm. In order to do so, the algorithm searched all *SNMI* entries within *SBs* reflecting the similarity between pairs of *REs* from different subjects to determine a global maximum. Two *REs* that were found to be related were seen as individual-level components but with the same underlying group-level component, also known as an aligned component (AC). These *RBs* were then searched to locate the local maxima within them as they indicate possible locations of the aligned component in different ICA realizations and subjects. Rows and columns containing these maxima were eliminated from the ***FSM*** when all *RCs* associated with the aligned component were located. This procedure was repeated until *c*_*max*_ = *max(c*_1 ≤ *f* ≤ *n*)_ *ACs* had been discovered, where *c* is the number of ICs and *f* (≤ *n*) is the number of subjects.

A *dn* × *dn* reproducibility matrix, ***M***_*rep*_, for each *AC* was then generated by collecting *NMIs* between all pairs of *REs* related to the *AC*. *NMIs* were used to provide a more straightforward interpretation of similarity. A maximum of one *RC* was selected per *AC* in each ICA realization to form its reproducibility matrix. Information contained within the reproducibility matrix was then divided into two metrics: inter-subject consistency and intra-subject reliability. Inter-subject consistency in this case was defined as the mean of all *NMIs* within inter-subject blocks. For a given *AC*, its consistency between subjects *i* and *m* can be calculated as:

(3) $$\begin{array}{l} \propto_{im}=mean\left(R_{i^{\prime }-m^{\prime }}\right)\\ =\frac{\sum_{j=1}^{D}\sum_{k=1}^{D}R_{ij-mk}\left[y\left(i,j\right),\;z\left(m,k\right)\right]}{K^{2}},\\ 1\leq i,m\leq N,i\neq m \end{array}$$

Equation (3) is representative of the mean NMI within the inter-subject block “*i*–*m*” in the reproducibility matrix, as it averages all the *NMI* values located at the intersection between realization *j* of subject *i* and realization *k* of subject *m*.

**Figure 5:**
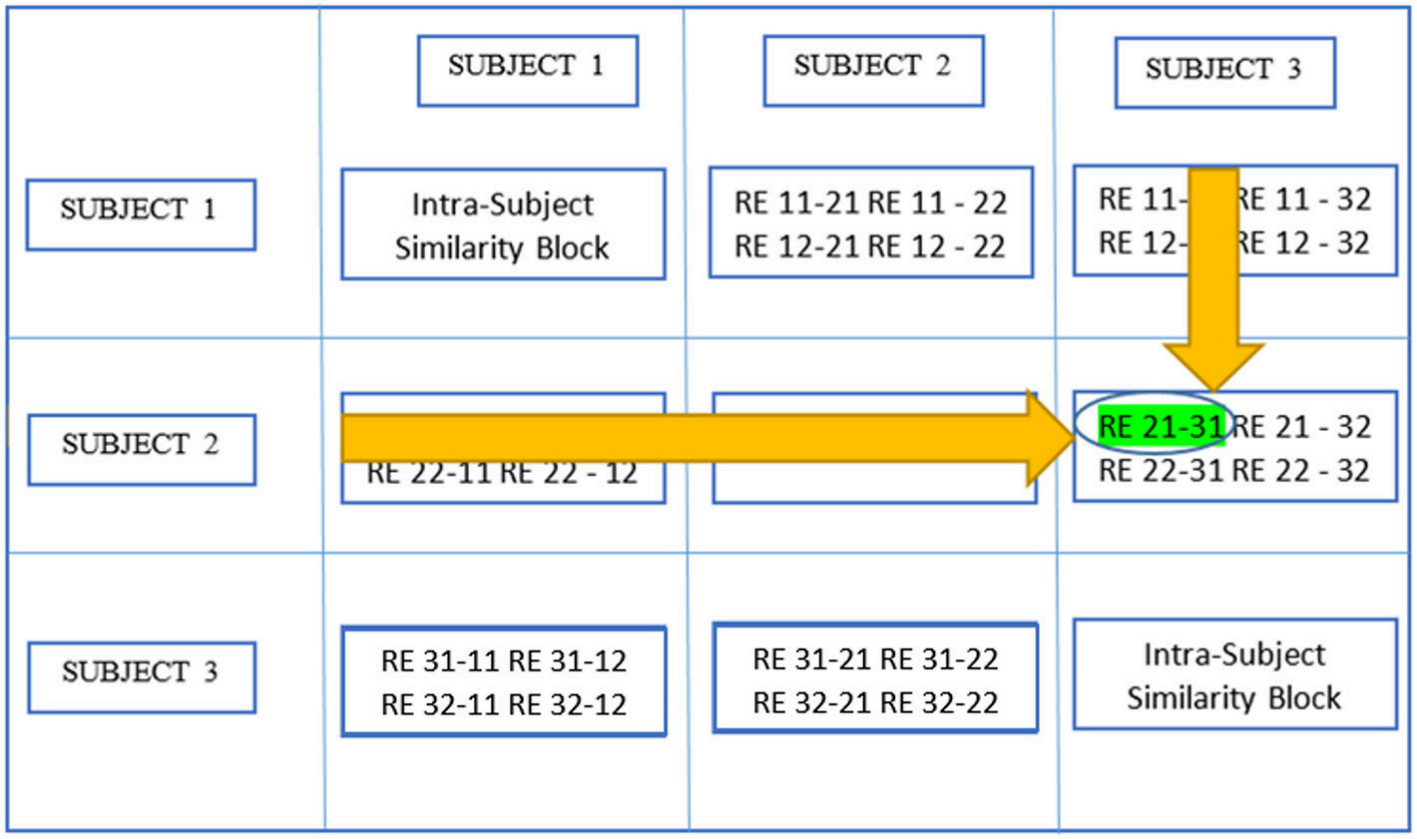
Determine global maximum. In this figure, the global maximum has a circle around it with highlighting.

Figure 5, which is a continuation of Figure 4, provides a demonstration of the third stage summarizing higher level stage description earlier using the same scenario as in the previous figure. The large circle mark represents a global maximum which is calculated by searching all *SNMIs* within the off-diagonal *SBs*. This enables compatibility with larger variations across subjects than within subjects. In this case, the global maximum was located at *R*_*ij*−*mk*_[*y, z*] which represents the *yth* row and the *zth* column of the *RB*. The two related *REs, RE*_*y*(*i,j*)_ and *RE*_*z*(*m,k*)_, are treated as individual-level components with the same underlying group-level component or *AC*. *RE*_*y*(*i,j*)_ represents the *yth* component of the *jth* realization of the *ith* subject.

**Figure 6:**
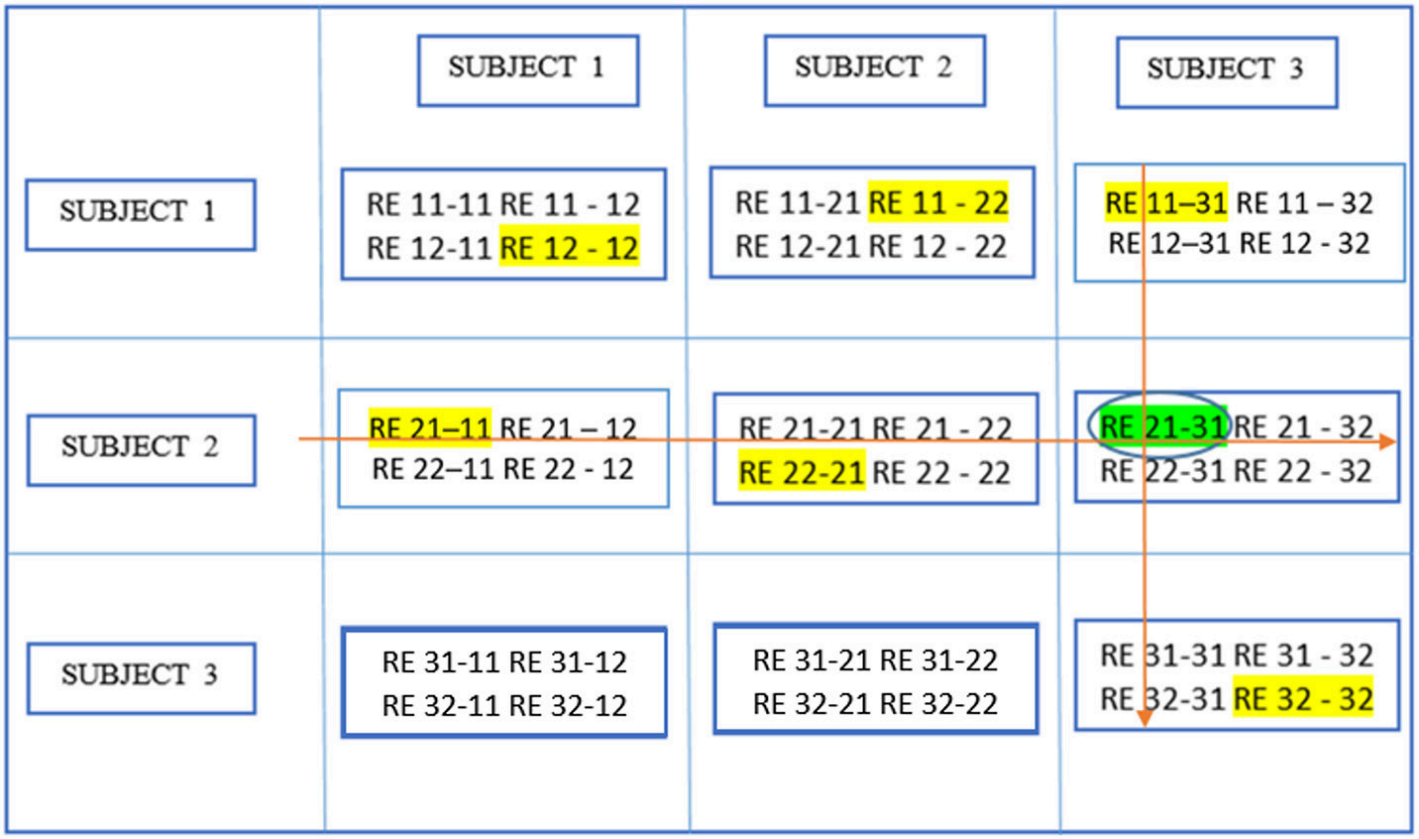
Determine block-wise maxima with highlighting in each block. Global maximum in this figure has a circle around it in addition and has been shown in an earlier figure as well.

Figure 6 demonstrates the next step which is to locate local maxima within these *RBs* by searching the *yth* rows of *RBs* $R_{ij-..}$ or all RBs containing *RE*_*y*(*i, j*)_, and the *zth* columns of *RBs* $R_{..-mk}$ or all *RBs* containing *RE*_*z*(*m, k*)_· This leads to the identification of the aligned component in different ICA realizations and subjects, *[y, v*_1_*]* or RE 21–11 in this example and *[u*_1_*, z]* or RE 11–31 where *u*_1_ and *v*_1_ are the relevant *RE* positions in individual *RBs* reflecting the largest similarity with *RE*_*y*(*i, j*)_ and *RE*_*z*(*m, k*)_ respectively. In this case, *u*_1_ = *v*_1_ means *[y, v*_1_*]* and *[u*_1_*, z]* pick up the same *RE* and the resulting component is thought of as pertaining to the aligned component determined by *RE*_*y*(*i,j*)_ and *RE*_*z*(*m,k*)_. If *u*_1_ and *v*_1_ are not equal, either *u*_1_ or *v*_1_ is picked based upon a voting procedure to determine the proximity of one or the other to more of those *REs* probed as the *u*_1_ = *v*_1_ case.

In the fourth and final stage of gRAICAR, we estimated *AC* maps and corresponding mixing time courses by using weighted averages of their related *REs*. To compute the weighted average of the *REs*, the first step is to define a subject load on inter-subject consistency representing the contribution or inter-subject centrality of a given subject to a given *AC* as follows:

(4) $$\begin{array}{l} \tau_{i\;}=\;\frac{1}{N-1}\;\sum_{m=1,\;m\neq i}^{N}\propto_{im\;}\;,\;1\;\leq i\;\leq \;N \end{array}$$

In (4), ∝_*im*_ refers to inter-subject consistency metric between subjects *i* and *m*. This equation can also be phrased as the inter-subject centrality of a subject in a given *AC* is the mean of the inter-subject consistency metrics between this subject and all others. The spatial maps and mixing time courses of an *AC* can be computed by combining this subject load on inter-subject consistency and the intra-subject reliability, as follows:

(5) $$\begin{array}{l} gIC_{n\;}=\;\frac{\sum_{i=1}^{N}\left[\beta_{i\;}\tau_{i}\sum_{j=1}^{D}RE_{p\left(i,j,n\right)}\right]\;}{K\sum_{i=1}^{N}\beta_{i\;}\tau_{i}},\;1\;\leq \;n\leq \;c_{max} \end{array}$$

*RE*_*p*(*i,j,n*)_ represents the *RE* or the spatial map of the IC as identified in the *jth* realization of the *ith* subject. *p* indexes the location of the *REs* and can vary with different realizations and subjects. The weights are different for each *AC*, computed by the *AC* specific reproducibility matrix.

*ACs* that were consistent across subjects were then statistically detected. The significance of cross-subject consistency of the resulting *AC* was explored using a two-step methodology. A non-parametric test was applied to select the *AC* consistent across all subjects. One *RE* from each ICA realization in the *FSM* was randomly sampled with replacement and the mean of inter-subject consistency metrics was computed after a non-participating subject was artificially generated. The aforementioned approach was very similar to the enhancement to the original RAICAR algorithm proposed by Yang et al. (2008). The aforementioned procedure was repeated 500 times. Resultant means of the inter-subject consistency metrics were combined to produce a null-distribution of inter-subject consistency. The 95th percentile, corresponding to a significance of *p* = 0.05, of the null distribution provided a threshold at this point. *ACs* with mean inter-subject consistency metrics greater than the aforementioned threshold value were regarded as common *ACs* across subjects. Null distributions of the subject loads on inter-subject consistency and intra-subject reliability for each one of the aforementioned *ACs* were generated by randomly assigning *REs* with replacement in the reproducibility matrix to artificially generated subjects. Corresponding to a significance value of *p* = 0.05, thresholds for the aforementioned metrics were then determined at 95th percentile of the corresponding null distributions. At this point, subjects above both of these threshold levels were considered to be representative of the *AC* under consideration. The main tasks pertaining to the fourth stage of gRAICAR algorithm were to estimate *AC* maps and corresponding time courses after weighted averaging their related *REs*, statistically detect *ACs* that were consistent across all subjects, and construct a graph for each *AC* for the characterization of relationships among subjects from an inter-subject consistency perspective.

### Clustering analysis

K-means algorithm has been previously used in fMRI analysis in several studies (Liu et al., 2012; Zhang and Li, 2012; Allen et al., 2014). We used this algorithm to examine the level of separation between Autism and TC groups for the ICs which were reproducible within each group, but not reproducible in the combined group. Clustering was unsupervised without using *a priori* subject groupings. We determined cluster purity per cluster as shown below:

(6) $$\begin{array}{l} Purity=\;\frac{1\;}{N\;}\;\sum_{i=1}^{k}max_{j}\;|\;c_{i\;}\cap \;t_{j}\;| \end{array}$$

In (6), *N* represents the number of data points or subjects, *k* the number of clusters, *c*_*i*_ the cluster in our analysis, and *t*_*j*_ the classification with maximum count for cluster *c*_*i*_.

Equation (7) shows our approach to determine sensitivity values where *SEN* represents sensitivity, *TP* true positive, and *FN* true negative.

(7) $$\begin{array}{l} SEN=\;\frac{TP}{TP+FN} \end{array}$$

Equation (8) shows our approach to determine specificity values where *SPC* represents specificity, *TN* true negative, and *FP* false positive.

(8) $$\begin{array}{l} SPC=\;\frac{TN}{TN+FP} \end{array}$$

### Analysis workflow

This section presents our implementation and workflow. For technical details and the rationale behind every step, we have included a technical discussion in earlier sections of this paper. We applied the gRAICAR algorithm thrice: first on the Autism group, second on the Control group, and then on the combined group. For the Autism and Control groups, we had 54 and 49 group-level components, respectively. For the combined (Autism + Control) group, we had 54 group-level components. We then examined these group-level components using criteria presented above describing the steps of gRAICAR algorithm and inter-subject consistency in (3). These criteria gave us 11 group-level components in the Autism group and 3 in the Control group. For all subjects, we accessed post-MELODIC analysis results and retrieved spatial maps associated with the ICs corresponding to each selected group-level component. MELODIC analysis was a part of data pre-processing and described earlier in this paper. We then processed these spatial maps in MATLAB wherein the spatial map associated with the IC index of the current subject was retrieved and singleton dimensions were removed. The resulting array was reshaped using MATLAB's reshape function (http://wwwmathworks.com/help/matlab/ref/reshape.html). thus giving us an *m* × *n* matrix where m is 1 for the current subject and n is 61 × 73 × 61 (=271,633) which was the size of each spatial map associated with the current IC index. After all subjects were processed, we had a 392 × 271,633 matrix for the Autism group and 407 × 271,633 matrix for the Control group. Suppose the resulting matrix for Autism is *A* while that for TC is *C*. We then combined *A* and *C* giving us a 799 × 271,633 matrix. We applied the k-means algorithm using this matrix to examine how subjects were clustered based on their spatial maps without *a priori* groupings. The aforementioned process was repeated for all permutations of group-level components selected based on pairing a component from the Autism group with one from the Control group resulting in 33 k-means clustering analysis (Autism Group: 11 × Control Group: 3). We had set up the algorithm to partition the data set into two clusters since we had two subject groups, Autism and Control. For each of these clustering permutations, the purity of clusters was identified based on how many subjects were correctly (or wrongly) clustered along with other subjects with the same diagnosis.

From the above analysis, the pair with maximum cluster purity was identified. Let the corresponding components be *A*^*x*^ and *C*^*x*^ in Autism and Control groups, respectively. The component in Controls with maximum spatial correlation with *A*^*x*^ (say, $C_{a}^{x}$) and the component in Autism with the maximum spatial correlation with *C*^*x*^ (say, $A_{c}^{x}$), were identified using the following approach:

(9) $$\begin{array}{l} \lambda =max\sum_{i=1}^{k}cov\left(\omega ,\omega_{i}\right) \end{array}$$

λ represents maximum covariance in (9), between group level component, ω, from the group being analyzed and ω_*i*_, that from the opposite group with *k* being the total number of group level components in the opposite group.

**Figure 7:**
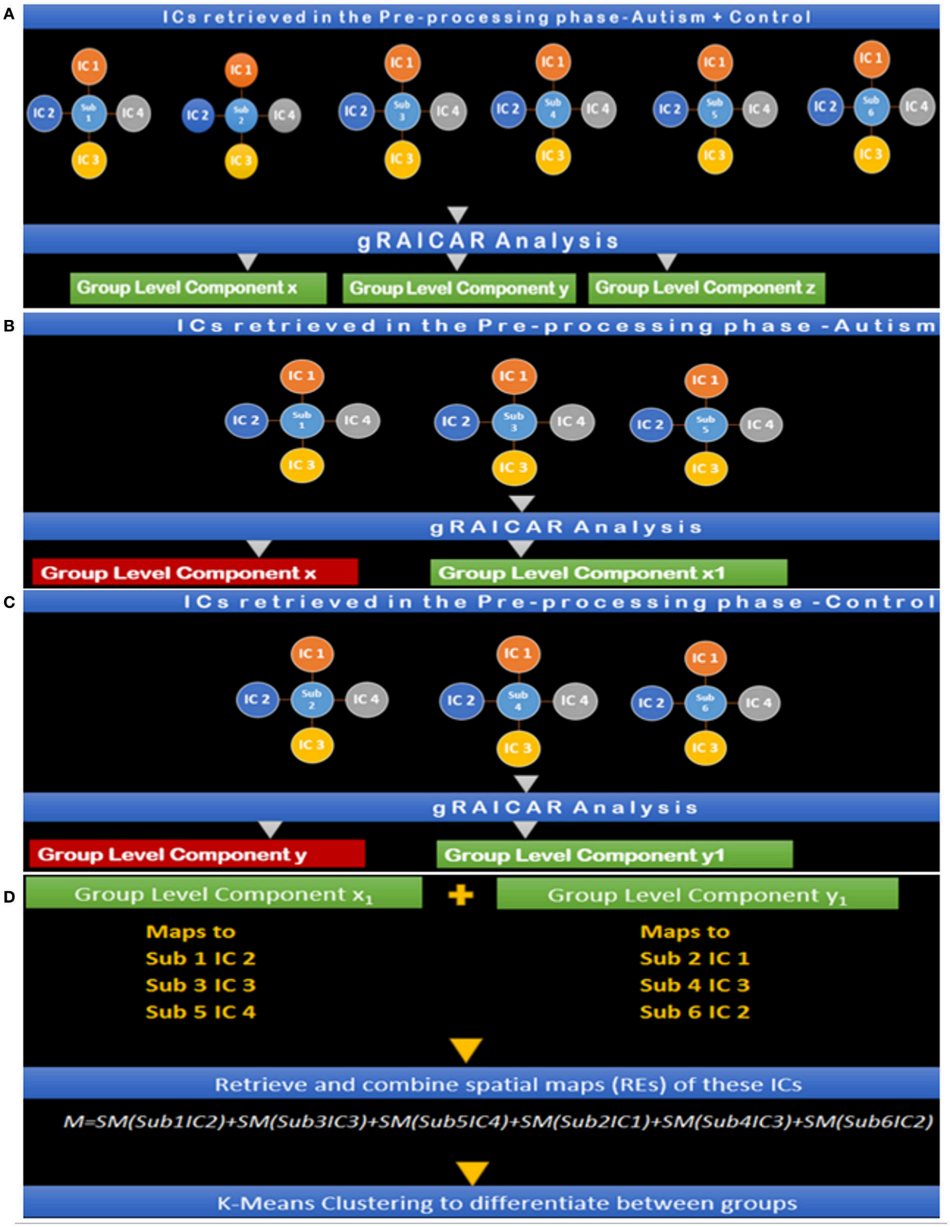
**(A)** Step I: gRAICAR Analysis on Combined (Autism + Control) Group producing group level components x, y, and z. **(B)** Step II: gRAICAR Analysis on Autism Group Only, producing group-level components, *x* and *x*_1_*. x* is discarded since it was produced in step I. **(C)** Step III: gRAICAR Analysis on Control Group Only, producing group-level components, *y* and *y*_1_. y is discarded since it was produced in step I. **(D)** Step IV: Group level components from Steps II and III were combined by retrieving spatial maps corresponding to ICs these group-level components represented for each subject within each group as shown in the figure. Group level components reproducible in the combined group also found in individual analysis (steps II and III) were excluded.

Two more k-means clustering analysis were performed by pairing *A*^*x*^ with $C_{a}^{x}$, and *C*^*x*^ with $A_{c}^{x}$. This analysis was carried out to ascertain whether the reproducible components in each group, when paired with the corresponding component with similar spatial distribution in the other group, can effectively discriminate between the groups. The entire analysis pipeline is illustrated in Figures 7A–D.

Steps I-IV presented in Figure 7 illustrate the concepts and summarize the processing by gRAICAR algorithm and k-means clustering analysis. For demonstration purposes, 6 artificial subjects, 3 from the Autism group (denoted by *A*) and the other 3 from Control group (denoted by *C*) are shown. Each example subject is assigned 4 ICs as shown. This is an arbitrary number illustrating the concept and the number of ICs was not constant in actual processing. In actual processing, 799 subjects were used with a variable number of ICs. (I) This step shows gRAICAR processing on all subjects in the combined group (Autism + Control groups) and the resulting list of group-level components, 3 in this case: *x, y*, and *z*. (II) This step shows gRAICAR analysis on Autism group only from the example and the list of group-level components obtained as in step I. Component *x* was found in step I as well and is discarded after visual examination. (II) This step shows gRAICAR analysis on Control group only and the list of group-level components obtained as in steps I and II. Component *y* was found in step I hence discarded. (IV) In this step, we completed multiple tasks. We combined the group-level components *x*_1_ and *y*_1_ by mapping these to individual ICs for each subject. We then retrieved spatial maps for each IC representing a subject under the group-level component and linearly combined them using MATLAB creating a matrix we called “***M***.” Finally, we used k-means clustering algorithm in MATLAB using ***M*** to investigate the separation of components between groups.

Once the clustering was complete, we constructed an inter-subject Euclidean distance matrix within both Autism and Control groups using spatial maps associated with each subject for component pairings (*C*^*x*^, *A*^*x*^), ($A_{c}^{x}$, *C*^*x*^), and ($C_{a}^{x}$, *A*^*x*^). A self-organizing map or SOM analyzes input vectors in the input space and learns, in an unsupervised manner, to classify them accordingly (Kohonen, 1988, 2001). The result includes a low-dimensional (one- or two-) discretized representation of the input space of the training samples referred to as a map.

Neighboring neurons in SOMs learn recognizing neighboring sections of the input space which leads them to not only learn the distribution but the topology of the training vectors used as input. These neurons are arranged in physical positions based upon a topology function and distances between them are calculated using a distance function.

Adjacent neurons in the topology generally are close in the input space as well. In our study, we used SOMs to visualize the reproducibility and separation of the subjects in feature-space in additional to the numerical values given by k-means. High dimensionality in k-means was scaled using SOMs for optimal visualization. We obtained individual spatial maps for *A*^*x*^ and *C*^*x*^ and stacked them into a matrix. We then used this matrix as input to a 5 × 5 SOM for visualization as described earlier. This process was repeated for (*A*^*x*^, $C_{a}^{x}$) and ($A_{c}^{x}$, *C*^*x*^).

## Results

Let us first examine the most reproducible group-level components within each group. We found 54 group-level components within the Autism group and 49 such components within the Control group. By combining selected components as described earlier, the range of cluster purity was 0.69–0.97 using unsupervised k-means clustering over all permutations of 11 group-level components from Autism and 3 from Controls. The average purity value was 0.89 with a standard deviation of 0.06.

**Figure 8:**
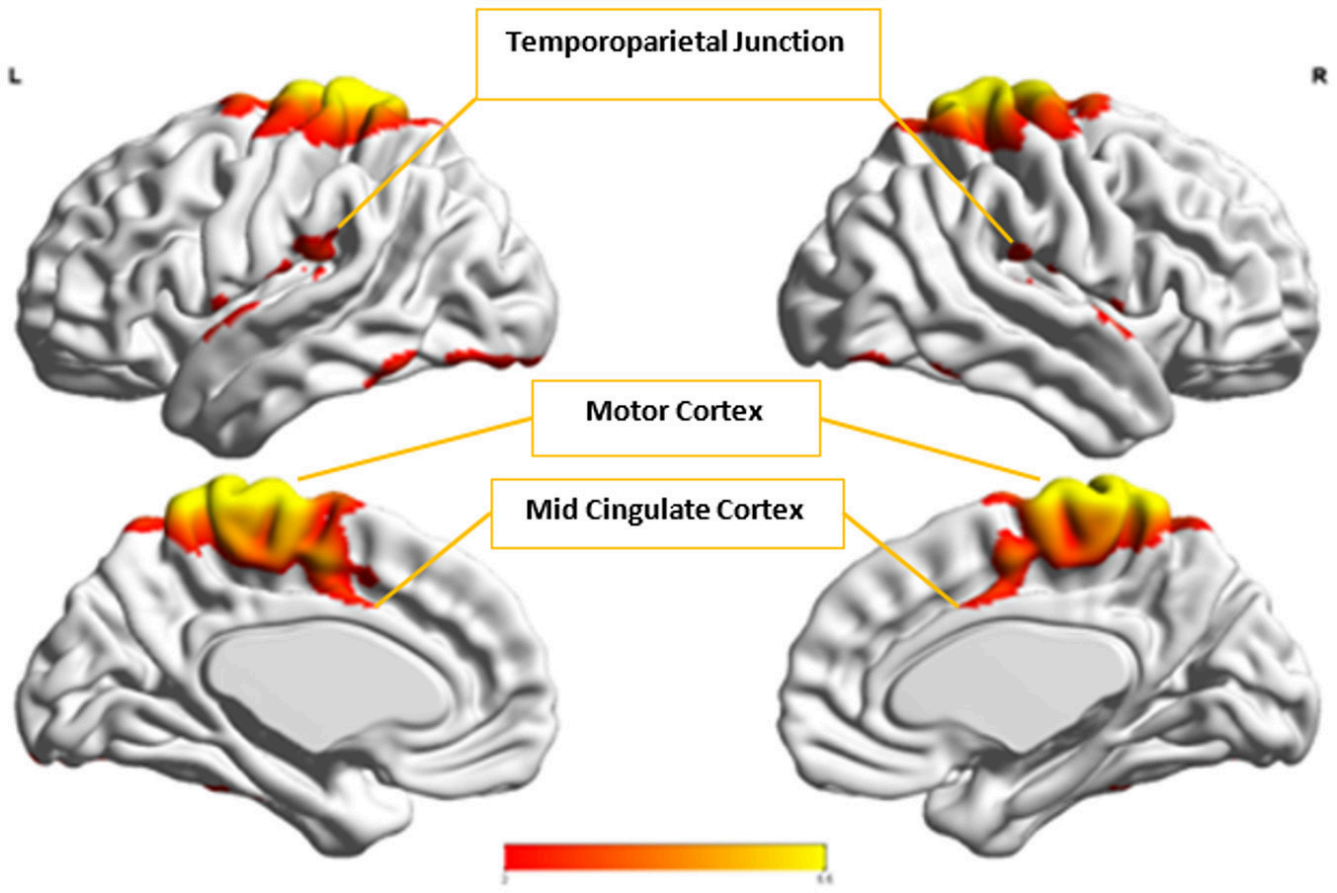
The group-level component, *A*^*x*^, from Autism group that produced the highest cluster purity value of 0.971 when combined with another group-level component from the Control group, *C*^*x*^.

**Figure 9:**
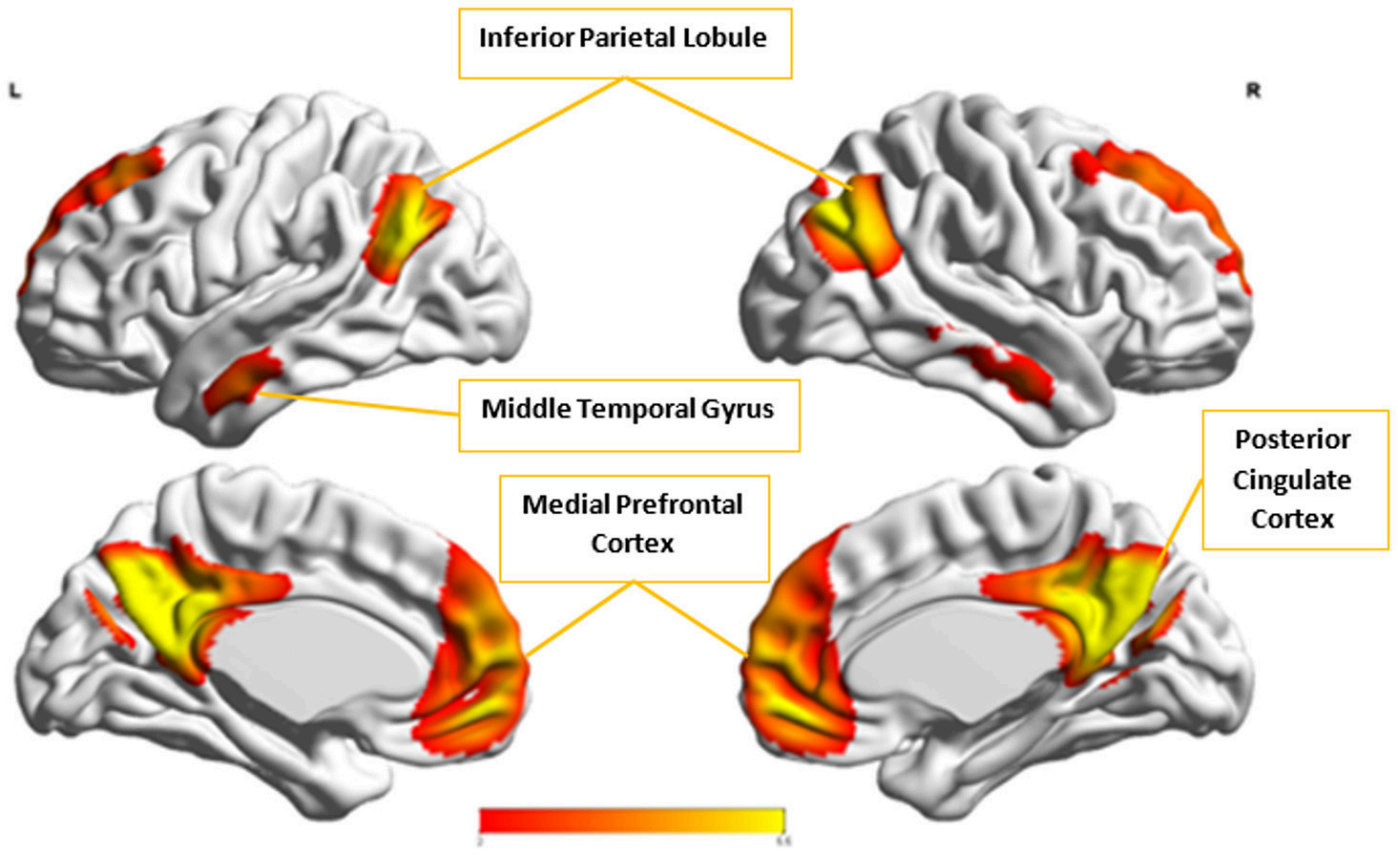
The group-level component, *C*^*x*^, from Control group that produced the highest cluster purity value of 0.971 when combined with another group-level component, *A*^*x*^, from the Autism group. These regions represent the default mode network (DMN).

**Figure 10:**
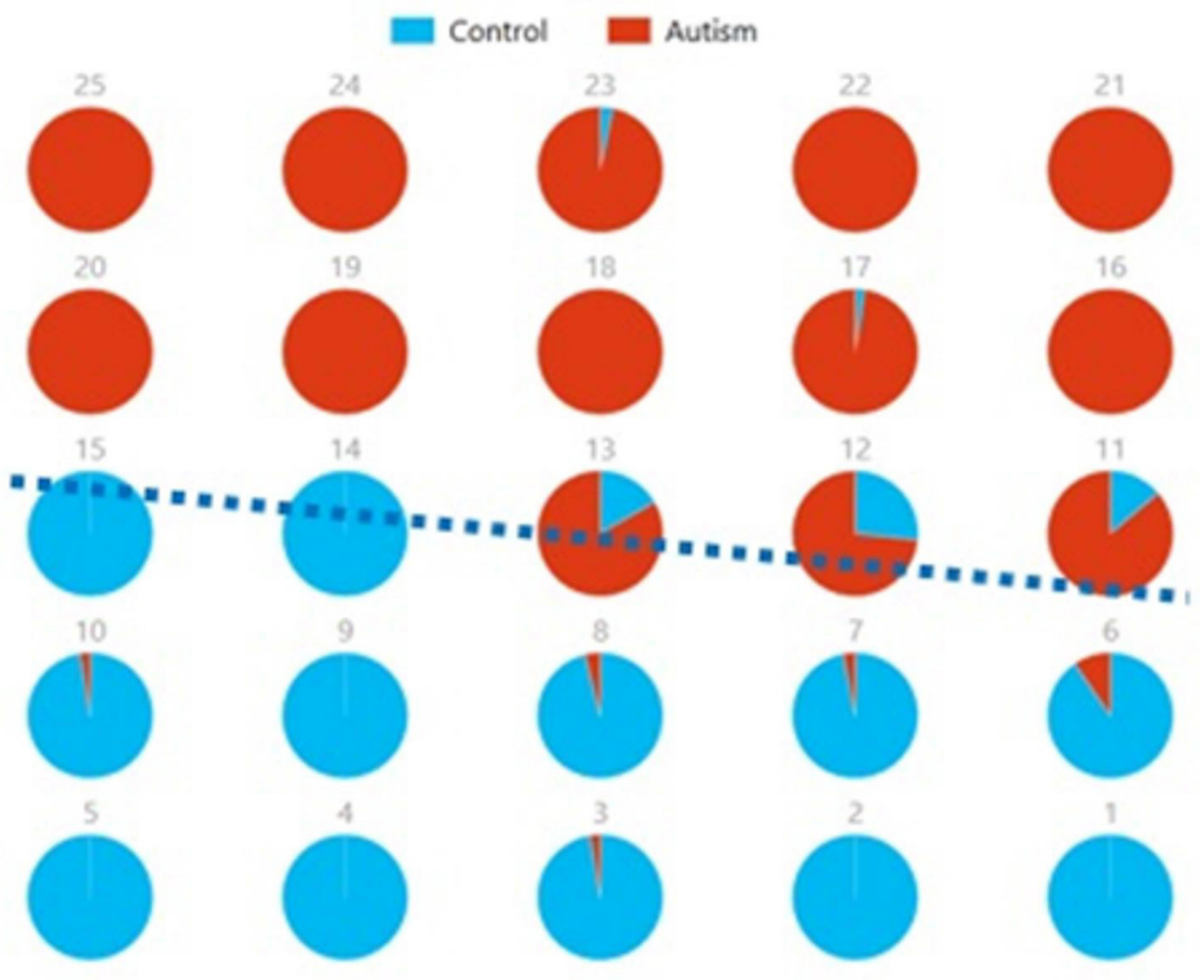
Pie chart visualizations based on a 5 × 5 SOM for A^*x*^ and C^*x*^ showing Autism and Control groups by neuron. Each pie chart corresponds to a neuron, represented by the number on each chart, in the SOM map for these components. This map indicates group separation approximately in the middle with Autism group populating the upper while Control the lower half of the SOM, represented by the dotted blue line.

Figures 8, 9 show the spatial maps of *A*^*x*^ and *C*^*x*^, respectively. The highest cluster purity value was 0.97 obtained by combining these two group-level components. Figure 10 presents a map of pie charts based on a 5 × 5 SOM to visualize the reproducibility and separation of the two groups using *A*^*x*^ and *C*^*x*^ as described earlier. Each pie chart represents the number of subjects from a given group, Autism or Control. As an example, a solid red chart represents all subjects from the Autism group whereas a solid blue all from the Control group.

**Figure 11:**
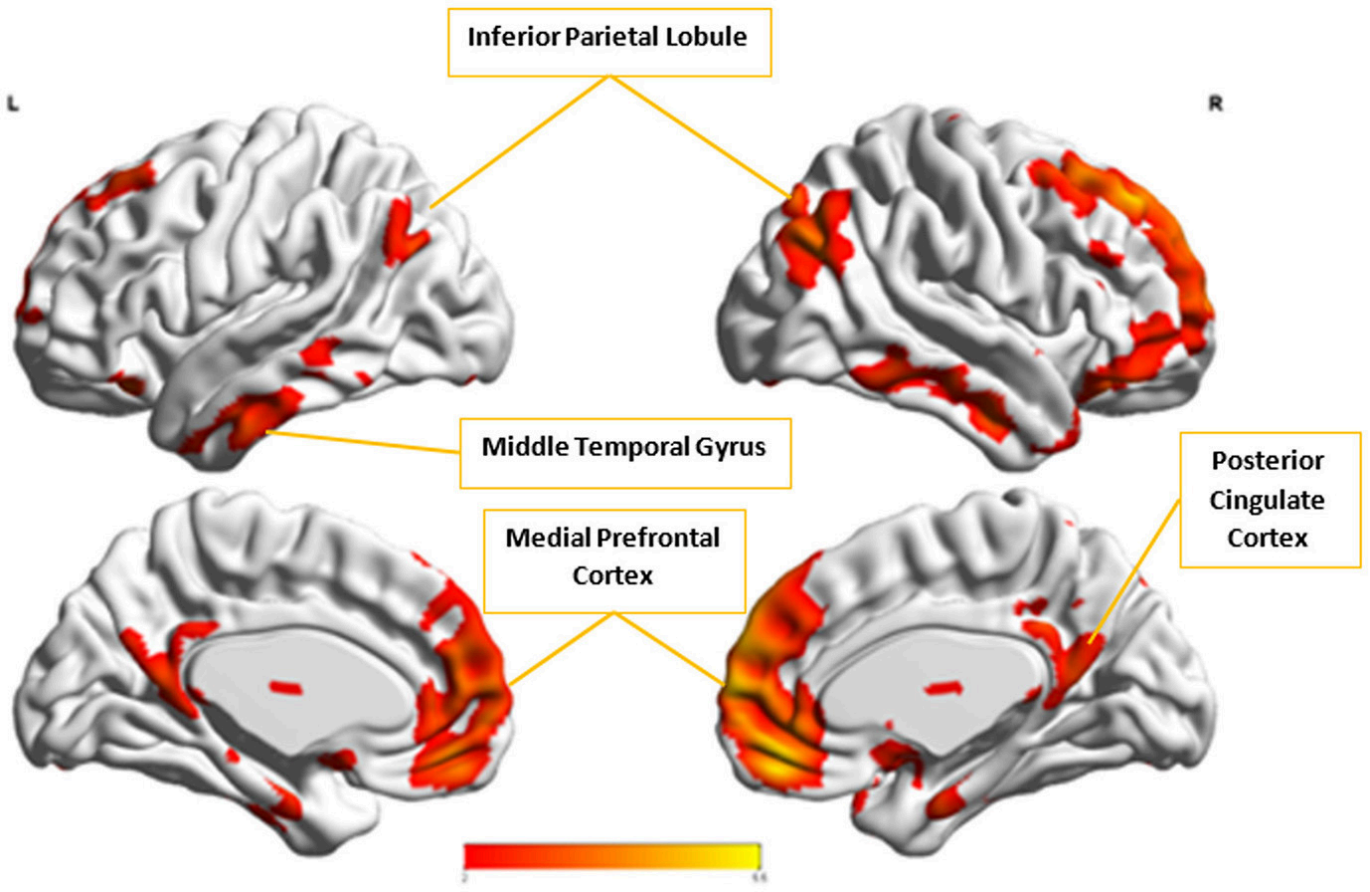
This figure represents the spatial map for $A_{c}^{x}$, the group-level component in the Autism group with the highest spatial correlation with *C*^*x*^. These regions represent the default mode network (DMN).

**Figure 12:**
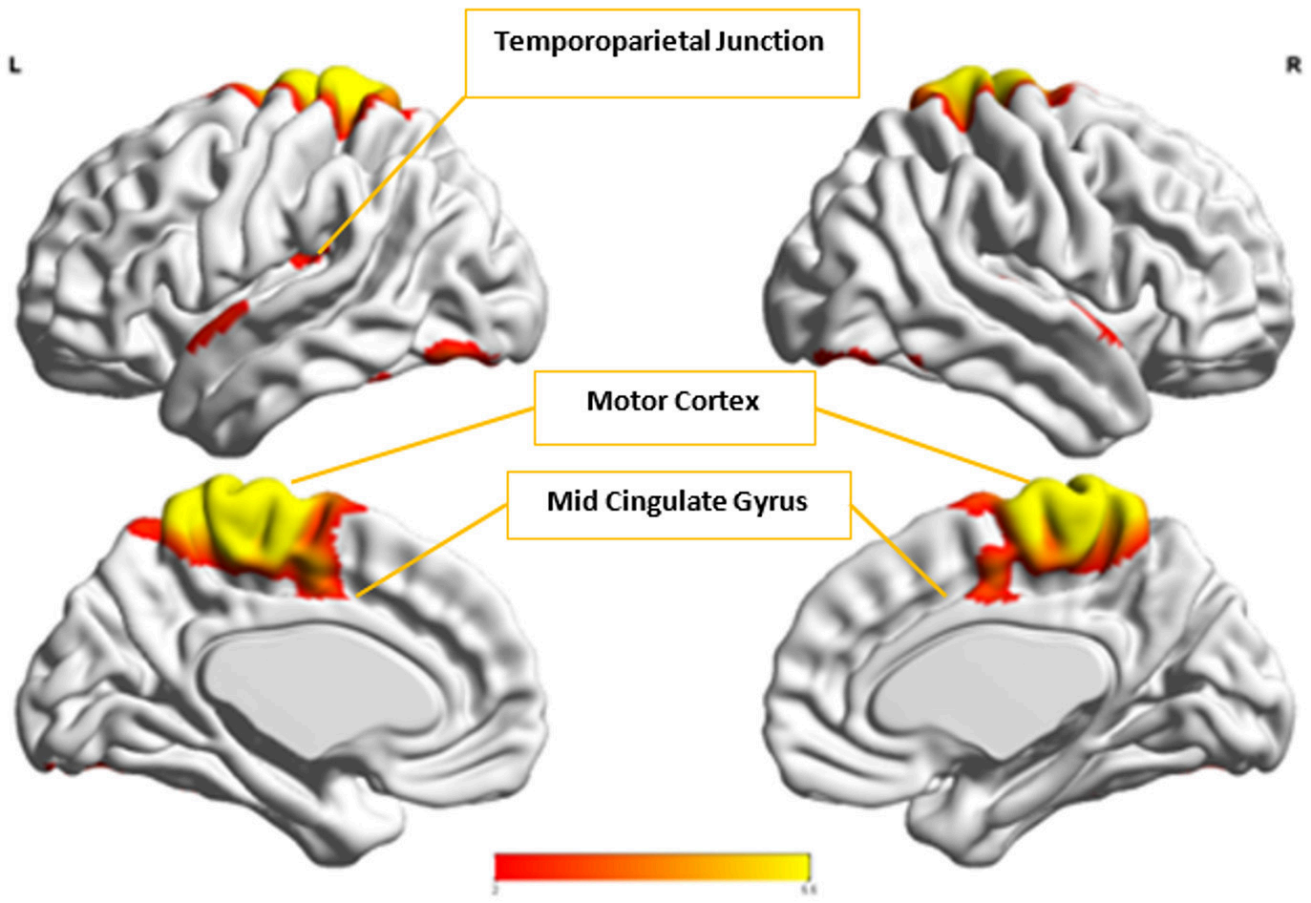
This figure represents the spatial map for $C_{a}^{x}$, the group-level component in the Control group with the highest spatial correlation with A^*x*^.

In the next step, we ascertained which group-level components in the opposite group had the highest spatial correlation to *A*^*x*^ and *C*^*x*^, using all 54 components from Autism and 49 from Control groups depending upon the comparison being carried out. *A*^*x*^ was found to have the highest spatial correlation value of 0.29 (*p* < 0.001) with $C_{a}^{x}$ in the Control group while *C*^*x*^ had the highest spatial correlation value of 0.63 (*p* < 0.001) with $A_{c}^{x}$ in the Autism group. We then combined *C*^*x*^ with $A_{c}^{x}$ and subjected them to k-means clustering analysis. This produced cluster purity of 0.895 with a sensitivity of 0.893 and a specificity of 0.897. Similarly, we combined *A*^*x*^ with $C_{a}^{x}$ as described earlier and completed k–means clustering analysis. This resulted in a cluster purity of 0.607 with a sensitivity of 0.43 and specificity of 0.77. Figures 11, 12 show the spatial profiles of $A_{c}^{x}$ and $C_{a}^{x}$, respectively.

**Figure 13:**
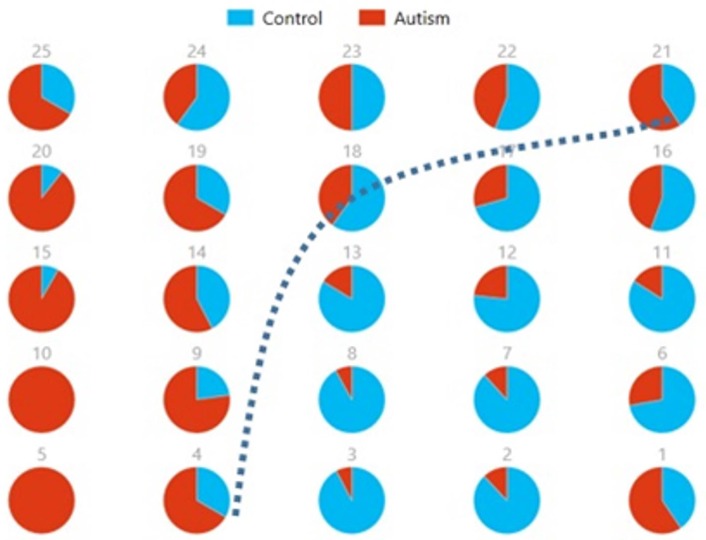
Pie chart visualizations based on a 5 × 5 SOM for *A*^*x*^ and $C_{a}^{x}$ showing Autism and Control groups by neuron where the number on each chart corresponds to a neuron in the SOM. The dotted line represents approximate separation between the two groups.

**Figure 14:**
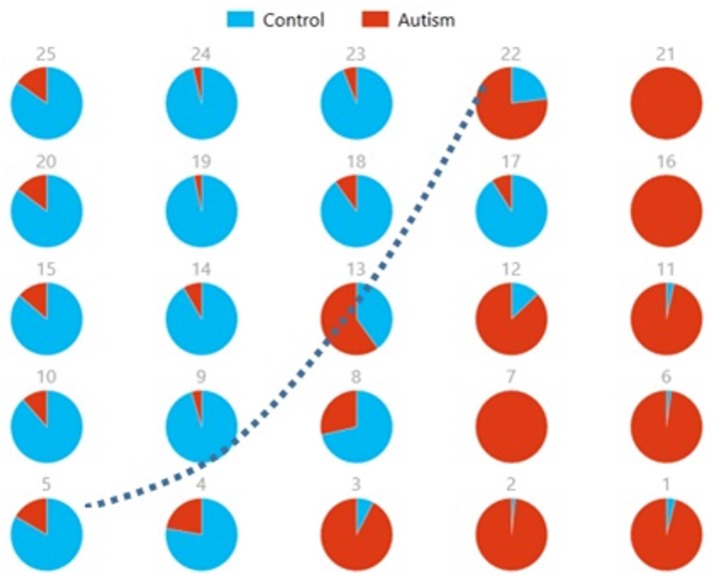
Pie chart visualizations based on a 5 × 5 SOM for $A_{c}^{x}$ and *C*^*x*^ showing Autism and Control groups by neuron where the number on each chart corresponds to a neuron in the SOM. The dotted line represents approximate separation between the two groups.

We also used pie charts to visualize the reproducibility and separation of subjects using 5 × 5 SOMs for these combinations: A^*x*^ + ${\text{C}}_{a}^{x}$ and C^*x*^ + ${\text{A}}_{c}^{x}$. These visualizations are presented in Figures 13, 14. In both cases, a dotted line represents the approximate separation observed between the two groups. Numbers on each pie chart represent the neuron in the SOM. It can be observed that the purity of individual pie charts drops when using spatial equivalents (Figures 13, 14) as compared to the most reproducible components in each group (Figure 10). This is a reflection of higher purity and separation in Figure 10 (97.1%) to lower purity values and hence lower separation in Figures 13, 14 (0.607 and 0.895 respectively).

## Discussion

We used a discover-confirm scheme wherein during the “discover” phase, we used gRAICAR to retrieve reproducible components in each group and during the “confirm” phase, we used unsupervised clustering to determine the separation between groups based on the reproducible components in each group. Further, the separation was visualized using self-organizing maps or SOMs. This is a novel methodological framework for investigating discriminative features between diagnostic groups as opposed to performing group-wise statistical tests or supervised classification.

Even though multiple studies have shown altered fMRI-based connectivity in certain brain networks in Autism using machine learning techniques, identifying individuals with Autism based on these measures has not been reliable especially in larger sized samples (Anderson et al., 2011; Plitt et al., 2015). We hypothesized that functional brain networks which are most reproducible separately within Autism and Control groups, but not reproducible when analyzing both groups as merged, may lead to effective discrimination between the groups. We tested the above hypothesis by finding the most reproducible ICA components (which represent brain networks) first in the merged and then in separate Autism and Control groups. Our results, shown in the previous section, indeed support the above hypothesis. SOM visualizations provided along with spatial maps of the group-level components give further insight into the reproducibility of certain brain networks as well as their differences between groups based on our proposition.

The overall cluster purity we obtained from our multisite fMRI data set, obtained by averaging the results obtained from the three scenarios was 0.824 with a sensitivity of 0.77 and specificity of 0.87. Previous studies using the same data set, but supervised classification methods instead of unsupervised clustering methods, have reported classification accuracies between 0.6 and 0.8 depending on whether they used a larger or smaller sub-sample of the ABIDE database (Anderson et al., 2011; Nielsen et al., 2013). Given the fact that the methods used here are different from the previous studies mentioned above, it would not be fair to directly compare our cluster purity with theirs. Instead, we would like to make the point that characterizing reproducibility of brain networks in different groups as well as the merged sample is a novel idea which may hold promise, especially in the context of disorders such as Autism. This is because the most discriminative features identified via the proposed method are more likely to be generalizable to a larger sample given the reproducibility constraint.

*C*_*x*_ and $A_{c}^{x}$, which provided highest discriminability between the groups, represent the default mode network (DMN) in Control and Autism groups, respectively. The DMN in Autism appears less prominent and incohesive. Decreased functional connectivity in default mode subnetworks contributes to core deficits observed in ASD patients (Assaf et al., 2010) whereas activity was reduced in the autism group in the ventral medial prefrontal cortex/ventral anterior cingulate cortex (Kennedy and Courchesne, 2008). Visuospatial working memory deficiency within the DMN was discovered in adolescents with ASD (Chien et al., 2016) and the regions of DMN functional connectivity in the bilateral inferior parietal lobule and posterior cingulate cortex were found to be smaller in ASD patients (Yasuhiro et al., 2016). On the other hand, *A*^*x*^ and $C_{a}^{x}$ represent regions of the motor network, mid cingulate cortex and temporal-parietal junction. Even though these regions have been implicated in autism (Chiu et al., 2008; Chantiluke et al., 2014; Kestemont et al., 2014; Nebel et al., 2014), it was not as discriminatory as the DMN. To summarize, our methodology first discovered highly reproducible components separately in Autism and Control groups pointing to functional networks described in this section. These components or functional networks they pointed to from both groups, when combined and analyzed in clustering analysis as described, provided high cluster purities, hence the ability to distinguish between the two groups. Functional networks discovered by applying our methodology separately in groups confirm earlier findings on alterations involving these networks in Autism. Results obtained from analyzing these networks support our hypothesis that functional networks highly reproducible separately in groups lead to higher cluster purities and discriminability.

### Limitations and future directions

Despite the fact that the ABIDE database provides invaluable means to analyze multisite resting state fMRI data sets with significant statistical power, there are certain inherent limitations to this data set. Site to site variability in acquisition parameters, subject populations, scanner performance, and research protocols may all be cofounding factors when it comes to the sensitivity for detecting abnormalities (Nielsen et al., 2013). It could be argued that the analysis of individual site data sets separately may provide a higher cluster purity. However, such results may be less easily translatable to the clinic because inter-site variability is something any potential clinical method will have to cope with. Both groups in ABIDE, Autism and healthy Control, appeared to have subjects with average to above-average range of IQ in addition to variation in diagnostic subtypes (Asperger's and PDD-NOS) across sites. A broader range of IQ levels need to be included in further studies since R-fMRI studies allow the inclusion of individuals with lower IQ than task based studies. In addition, not all sites spanned childhood to middle adulthood but further studies can include a deeper examination of the development of brain providing insight into developmental dynamics of Autism (Di Martino et al., 2014).

We used a novel analysis framework involving gRAICAR as described earlier (Yang et al., 2012). Despite its robustness, there are several limitations including computational and physical memory costs. We were able to mitigate computational and physical memory concerns by using parallel processing and cloud computing. gRAICAR further provides the ability to parallelize one of the processing stages hence reducing the computational time and increasing efficiency. We had used gRAICAR code in a UNIX/MATLAB environment. Also in the absence of a threshold in gRAICAR to determine the existence of a relationship, the RCs are forced to align with a group-level component even if there is low similarity with other RCs. In future studies, it would be interesting to investigate how gRAICAR performs in site-level analytics within Autism and ABIDE data sets. Our methodology can also be expanded to other neurological disorders to determine the utility of this algorithm in future studies.

## Author contributions

MS: Main author responsible for data acquisition, preparation, methodology implementation, post-processing, visualizations, documenting results and paper writing work. ZY: Introduced and published the methodology implemented in the paper, research and editing consultant. XH: Methodology contributor, editing consultant. GD: Principal Investigator and project scientist, principal editor.

### Conflict of interest statement

The authors declare that the research was conducted in the absence of any commercial or financial relationships that could be construed as a potential conflict of interest.

## References

[B1] Allen E. Damaraju E. Plis S. Erhardt E. Eichele T. Calhoun V. (2014). Tracking whole-brain connectivity dynamics in the resting state. Cereb. Cortex 24, 663–676. 10.1093/cercor/bhs352 23146964 PMC3920766

[B2] American Psychiatric Association (2013). Diagnostic and Statistical Manual of Mental Disorders, Arlington, VA: American Psychiatric Publishing.

[B3] Anderson J. Nielsen J. Froehlich A. DuBray M. Druzgal T. Cariello A.. (2011). Functional connectivity magnetic resonance imaging classification of Autism. Brain 134, 3742–3754. 10.1093/brain/awr263 22006979 PMC3235557

[B4] Assaf M. Jagannathan K. Calhoun V. Miller L. Stevens M. Sahl R.. (2010). Abnormal functional connectivity of default mode sub-networks in autism spectrum disorder patients. Neuroimage 53, 247–256. 10.1016/j.neuroimage.2010.05.067 20621638 PMC3058935

[B5] Baio J. (2014). Prevalence of autism spectrum disorder among children aged 8 years - autism and developmental disabilities monitoring network, 11 Sites, United States, 2010. Morbid. Mort. Weekly Rep. (MMWR) 63, 1–21. Available online at: https://www.cdc.gov/mmwr/pdf/ss/ss6302.pdf 24670961

[B6] Beckman C. DeLuca M. Devlin J. Smith S. (2005). Investigations into resting-state connectivity using independent component analysis. Philos. Trans. R. Soc. 360, 1001–1013. 10.1098/rstb.2005.1634 PMC1854918 16087444

[B7] Beckman C. Smith S. (2004). Probabilistic independent component analysis. IEEE Trans. Med. Imaging 23, 137–152. 10.1109/TMI.2003.822821 14964560

[B8] Bell A. Sejnowski T. (1995). An information-maximization approach to blind separation and blind deconvolution. Neural Comput. 7, 1129–1159. 10.1162/neco.1995.7.6.1129 7584893

[B9] Calderoni S. Retico A. Biagi L. Tancredi R. Muratori F. Tosetti M. (2012). Female children with autism spectrum disorder: an insight from mass-univariate and pattern classification analyses. Neuroimage 59, 1013–1022. 10.1016/j.neuroimage.2011.08.070 21896334

[B10] Chantiluke K. Barrett N. Giampietro V. Santosh P. Brammer M. Simmons A.. (2014). Inverse fluoxetine effects on inhibitory brain activation in non-comorbid boys with ADHD and with ASD. Psychopharmacology 232, 2071–2082. 10.1007/s00213-014-3837-2 25533997 PMC4432080

[B11] Chien H. Y. Gau S. Isaac S. Tseng W. Y. (2016). Deficient visuospatial working memory functions and neural correlates of the default-mode network in adolescents with autism spectrum disorder. Autism Res. 9, 1058–1072. 10.1002/aur.1607 26829405

[B12] Chiu P. Kayali M. Kishida K. Tomlin D. Klinger L. Klinger M.. (2008). Self responses along cingulate cortex reveal quantitative neural phenotype for high-functioning autism. Neuron 57, 463–473. 10.1016/j.neuron.2007.12.020 18255038 PMC4512741

[B13] Coutanche M. Thompson-Schill S. Schultz R. (2011). Multi-voxel pattern analysis of fMRI data predicts clinical symptom severity. Neuroimage 57, 113–123. 10.1016/j.neuroimage.2011.04.016 21513803 PMC3105443

[B14] Deshpande G. Libero L. Sreenivasan K. Deshpande H. Kana R. (2013). Identification of neural connectivity signatures of autism using machine learning. Front. Hum. Neurosci. 7:670. 10.3389/fnhum.2013.00670 24151458 PMC3798048

[B15] Di Martino A. Yan C. Li Q. Denio E. Castellanos F. Alaerts K.. (2014). The autism brain imaging data exchange: towards a large-scale evaluation of the intrinsic brain architecture in autism. Mol. Psychiatry 19, 659–667. 10.1038/mp.2013.78 23774715 PMC4162310

[B16] Ecker C. Marquand A. Mourão-Miranda J. Johnston P. Daly E. M. Brammer M. J.. (2010). Describing the brain in autism in five dimensions–magnetic resonance imaging-assisted diagnosis of autism spectrum disorder using a multiparameter classification approach. J. Neurosci. 30, 10612–10623. 10.1523/JNEUROSCI.5413-09.2010 20702694 PMC6634684

[B17] Greicius M. Krasnow B. Reiss A. Menon V. (2003). Functional connectivity in the resting brain: a network analysis of the default mode hypothesis. Proc. Natl. Acad. Sci. U.S.A. 100, 253–258. 10.1073/pnas.0135058100 12506194 PMC140943

[B18] Jenkinson M. Beckmann C. Behrens T. Woolrich M. Smith S. (2012). the FMRIB Software Library (FSL). Neuroimage 62, 782–790. 10.1016/j.neuroimage.2011.09.015 21979382

[B19] Jiao Y. Chen R. Ke X. Chu K. Lu Z. Herskovits E. H. (2010). Predictive models of autism spectrum disorder based on brain regional cortical thickness. Neuroimage 50, 589–599. 10.1016/j.neuroimage.2009.12.047 20026220 PMC2823830

[B20] Kennedy D. Courchesne E. (2008). Functional abnormalities of the default network during self- and other-reflection in autism. Soc. Cogn. Affect. Neurosci. 3, 177–190. 10.1093/scan/nsn011 19015108 PMC2555458

[B21] Kestemont J. Ma N. Baetens K. Clément N. Van Overwalle F. Vandekerckhove M. (2014). Neural correlates of attributing causes to the self, another person and the situation. Soc. Cogn. Affect. Neurosci. 10, 114–121. 10.1093/scan/nsu030 24633532 PMC4994850

[B22] Kohonen T. (1988). Self-Organization and Associative Memory 8. Heidelberg: Springer-Verlag.

[B23] Kohonen T. (2001). Self-Organizing Maps, 3rd Edn. Heidelberg: Springer-Verlag.

[B24] Kraskov A. Stögbauer H. Grassberger P. (2004). Estimating mutual information. Phys. Rev. Stat. Nonl. Soft Matter Phys. 69:066138. 10.1103/PhysRevE.69.066138 15244698

[B25] Libero L. DeRamus T. Lahti A. Deshpande G. Kana R. (2015). Multimodal neuroimaging based classification of Autism Spectrum Disorder using anatomical, neurochemical and white matter correlates. Cortex 66, 46–59. 10.1016/j.cortex.2015.02.008 25797658 PMC4782775

[B26] Liu W. Awate S. Fletcher P. (2012). Group analysis of resting-state fMRI by hierarchical markov random fields, in Medical Image Computing and Computer-Assisted Intervention - Lecturer Notes in Computer Science (Nice). 10.1007/978-3-642-33454-2_24 PMC3749875 23286130

[B27] Maes F. Collignon A. Vandermeulen D. Marchal G. Suetens P. (1997). Multimodality image registration by maximization of mutual information. IEEE Trans. Med. Img 16, 187–198. 10.1109/42.563664 9101328

[B28] Maximo J. Keown C. Nair A. Müller R. (2013). Approaches to local connectivity in autism using resting state functional connectivity MRI. Front. Hum. Neurosci. 7:605. 10.3389/fnhum.2013.00605 24155702 PMC3792552

[B29] Muhle R. Trentacoste S. V. Rapin I. (2004). The genetics of Autism. Pediatrics 113 e472-86. 10.1542/peds.113.5.e472 15121991

[B30] Nebel M. Eloyan A. Barber A. Mostofsky S. (2014). Precentral gyrus functional connectivity signatures of autism. Front. Syst. Neurosci. 8:80. 10.3389/fnsys.2014.00080 24860442 PMC4030180

[B31] Nielsen J. Zielinski B. Fletcher P. Alexander A. Lange N. Bigler E.. (2013). Multisite functional connectivity MRI classification of autism: ABIDE results. Front. Hum. Neurosci. 7:599. 10.3389/fnhum.2013.00599 24093016 PMC3782703

[B32] Ozonoff S. Young G. S. Carter A. Messinger D. Yirmiya N. Zwaigenbaum L.. (2011). Recurrence risk for autism spectrum disorders: a Baby Siblings Research Consortium study. Pediatrics 128, e488–e495. 10.1542/peds.2010-2825 21844053 PMC3164092

[B33] Plitt M. Kelly A. Martina A. (2015). Functional connectivity classification of autism identifies highly predictive brain features but falls short of biomarker standards. Neuroimage Clin. 7, 359–366. 10.1016/j.nicl.2014.12.013 25685703 PMC4309950

[B34] Pluim J. Maintz J. Viergever M. (2003). Mutual-information-based registration of medical images: a survey. EEE Trans. Med. Imaging 22, 986–1004. 10.1109/TMI.2003.815867 12906253

[B35] Sato J. R. Hoexter M. Q. Oliveira P. P. Brammer M. J. MRC AIMS Consortium Murphy C.. (2013). Inter-regional cortical thickness correlations are associated with autistic symptoms: a machine-learning approach. J. Psychiatr. Res. 47, 453–459. 10.1016/j.jpsychires.2012.11.017 23260170

[B36] Song X. Dong Z. Long X. Li S. Zuo X. Zhu C.. (2011). REST: a toolkit for resting-state functional magnetic resonance imaging data processing. PLoS ONE 6:e25031. 10.1371/journal.pone.0025031 21949842 PMC3176805

[B37] Supekar K. Uddin L. Khouzam A. Phillips J. Gaillard W. Kenworthy L. (2013). Brain hyper-connectivity in children with autism and its links to social deficits. Cell Reports 5, 738–747. 10.1016/j.celrep.2013.10.001 24210821 PMC3894787

[B38] Uddin L. Q. Menon V. Young C. B. Ryali S. Chen T. Khouzam A.. (2011). Multivariate searchlight classification of structural magnetic resonance imaging in children and adolescents with autism. Biol. Psychiatry 70, 833–841. 10.1016/j.biopsych.2011.07.014 21890111 PMC3191298

[B39] von dem H. E. Stoyanova R. Baron-Cohen S. Calder A. (2013). Reduced functional connectivity within and between ‘social’ resting state networks in autism spectrum conditions. Soc. Cogn. Affect. Neurosci. 8, 694–701. 10.1093/scan/nss053 22563003 PMC3739917

[B40] Wang H. Chen C. Fushing H. (2012). Extracting multiscale pattern information of fMRI based functional brain connectivity with application on classification of autism spectrum disorders. PLoS ONE 7:e45502. 10.1371/journal.pone.0045502 23056205 PMC3466274

[B41] Wingate M. Mulvihill B. Kirby R. S. Pettygrove S. Cunniff C. Meaney F. (2012). Prevalence of autism spectrum disorders–Autism and Developmental Disabilities Monitoring Network, 14 sites, United States, 2008. Morbid. Mort. Weekly Rep. 61, 1–19. Available online at: https://www.cdc.gov/mmwr/pdf/ss/ss6103.pdf 22456193

[B42] Woolrich M. Jbabdi S. Patenaude B. Chappell M. Makni S. Behrens T.. (2009). Bayesian analysis of neuroimaging data in FSL. Neuroimage 45(Suppl.), S173–S186. 10.1016/j.neuroimage.2008.10.055 19059349

[B43] Yan C. Zang Y. (2010). DPARSF: A MATLAB Toolbox for “Pipeline” data analysis of resting-state fMRI. Front. Syst. Neurosci. 4:13 10.3389/fnsys.2010.00013 20577591 PMC2889691

[B44] Yang Z. Chang C. Xu T. Jiang L. Handwerker D. Castellanos F.. (2014a). Connectivity trajectory across lifespan differentiates the precuneus from the default network. NeuroImage 89, 45–56. 10.1016/j.neuroimage.2013.10.039 24287438 PMC3944140

[B45] Yang Z. LaConte S. Weng X. Hu X. (2008). Ranking and averaging independent component analysis by reproducibility (RAICAR). Hum. Brain Mapp. 29, 711–725. 10.1002/hbm.20432 17598162 PMC6870671

[B46] Yang Z. Xu Y. Xu T. Hoy C. Handwerker D. Chen G.. (2014b). Brain network informed subject community detection in early-onset schizophrenia. Sci. Rep. 4:5549. 10.1038/srep05549 24989351 PMC4929688

[B47] Yang Z. Zuo X. Wang P. Li Z. LaConte S. Bandettini P.. (2012). Generalized RAICAR: discover homogeneous subject (sub)groups by reproducibility of their intrinsic connectivity networks. Neuroimage 63, 403–414. 10.1016/j.neuroimage.2012.06.060 22789741

[B48] Yasuhiro F. Masafumi H. Hideki O. Kenji M. Hiromichi I. Takashi I. (2016). Default mode network abnormalities in children with autism spectrum disorder detected by resting-state functional magnetic resonance imaging. J. Med. Invest. 63, 204–208. 10.2152/jmi.63.204 27644559

[B49] Zhang S. Li C. (2012). Functional connectivity mapping of the human precuneus by resting state fMRI. NeuroImage 59, 3548–3562. 10.1016/j.neuroimage.2011.11.023 22116037 PMC3288461

